# Aberrant Non-Coding RNA Expressed in Gastric Cancer and Its Diagnostic Value

**DOI:** 10.3389/fonc.2021.606764

**Published:** 2021-07-06

**Authors:** Zhilong Yu, ZeYin Rong, Jinxin Sheng, Zai Luo, Jianming Zhang, Tengfei Li, Zhonglin Zhu, Zhongmao Fu, Zhengjun Qiu, Chen Huang

**Affiliations:** ^1^ Department of General Surgery, Shanghai General Hospital, School of Medicine, Shanghai Jiao Tong University, Shanghai, China; ^2^ Department of General Surgery, Haimen People’s Hospital, Haimen, China

**Keywords:** gastric cancer, noncoding RNAs, biomarker, microarray, diagnosis

## Abstract

Gastric cancer (GC) is one of the digestive tract malignancies with high invasion and mortality rates. Recent studies have reported that non-coding RNAs (ncRNAs) seem to play a crucial part in many tumors. Due to their high stability, ncRNAs may used as novel biomarkers to predict the occurrence and prognosis of GC. Here, we measured miRNA, lncRNA and cirRNA expression profiles of GC patients by using microarray and RNA-sequencing data from tissue samples. The diagnosis prediction model based on the ncRNA signatures and clinical features was evaluated by circulating and tissue validation and ROC analysis. Nine miRNAs and eight lncRNAs were obtained from the microarray analysis. Six miRNAs (miR-550a-5p, miRNA-936, miR-1306-3p, miR-3185, miR-6083, miR-6792-3p) and three lncRNAs (lnc-MB21D1-3:5, lnc-PSCA-4:2 and lnc-ABCC5-2:1) were abnormally expressed in circulating and tissue samples compared with normal control (NC), which was closely related to clinical pathology and survival time of GC patients; circRNA sequencing and qRT-PCR revealed four circRNAs (circASHL2, circCCDC9, circNHSL1 and cirMLLT10) were abnormally expressed in GC tissues and parts of them were negative relationship with their predicted binding miRNAs. These ncRNAs might act as promising molecular markers for the diagnosis and prognosis of gastric cancer.

## Introduction

Gastric cancer (GC) is the sixth among the most common malignancies and the second leading cause of deaths by cancer in the world, with nearly 1,033,701 new cases and 782,685 deaths in 2018 (1). The recent cancer statistics in China demonstrated that GC is ranked the second in new cancer occurrence amount and the third leading cause for cancer mortality (2). Without obvious initial symptoms, most of GC patients were diagnosed with advanced gastric carcinoma. In spite of the improvement of surgery, chemotherapy, radiotherapy and targeted therapy strategies, the overall prognosis for GC patients remained dissatisfactory (3, 4). Nowadays, gastroscopy is still the golden criterion for diagnosing gastric cancer. In part of East Asian countries, like Japan and Korea, they carried out a screening program based on endoscopy to detect early gastric cancer for many years. The rate of early detection of GC had increased to 50% by 2009 due to government-sponsored screening programs. Therefore, 5 year survival of GC patients is high in Japan (64.6%) and South Korea (71.5%) (5, 6). However, endoscopy screening is too expensive and invasive to be popularized in China. Thus, noninvasive or minimally invasive markers are widely used in clinical. Currently, the traditional GC-associated serologic markers, like carcinoembryonic antigen (CEA), carbohydrate antigen 19-9 (CA19-9), carbohydrate antigen 50 (CA50), and carbohydrate antibody 72-4 (CA72-4), had no adequate sensitivity and specificity to achieve early detection (7–9). As a result, it is urgent to further develop novel biomarkers with high potential clinical value for early detection and to improve the prognosis of patients for gastric carcinoma.

Noncoding RNAs (ncRNAs) always be divided into microRNAs (miRNAs), long noncoding RNAs (lncRNAs), circular RNAs (circRNAs), pRNA, and tRNA. Among the ncRNAs, miRNAs are the most studied, they can transcriptionally regulated gene expression by repression of the target mRNA with 22 nucleotides in length. Multiple studies have demonstrated that miRNA was stable and could be easily detected in different tissue, blood, feces, saliva and ascites (10–12).

Compared to miRNAs, lncRNAs, which are 200 nt-100kb long transcripts are less conserved (13, 14). Recent studies indicated that the relationship between lncRNA and miRNA was complicated, and a regulatory network came into being, in which lncRNA affected miRNA levels, miRNA triggered lncRNA decay, and lncRNA competed with miRNA for mRNA interaction (15–17). Except for these linear miRNAs and lncRNAs, circRNAs, which are a kind of novel noncoding RNA without 5′ caps or 3′ tails (18). Recently, emerging evidence indicated that circRNAs might play a crucial role in cancer, in which they could be served as competing endogenous (ceRNA) RNA to compete for miRNA-binding sites by sponging miRNA (19, 20). Accumulating increasing evidence indicated that ncRNAs were widely involved in various cancer, especially GC (21–23). However, a comprehensive and in-depth research of ncRNAs in GC has not been reported, which is worthy of further investigation.

In this study, we investigated the potential use of circulating ncRNAs in plasma as biomarkers of GC. First, we identified aberrant expression of ncRNAs by microarray and bioinformatics approaches which included GO and KEGG enrichment analysis. Second, we employed quantitative reverse transcription polymerase chain reaction (qRT-PCR) to confirm and validate the selected ncRNAs signatures and clinical features. Furthermore, receiver operator characteristic (ROC) curve analysis was run to evaluate the diagnostic value of the differentially expressed ncRNAs as biomarkers of GC. Ultimately, we explored these ncRNAs survival curves based on their expression level. The result revealed that these differentially expressed ncRNAs might serve as novel biomarkers for the diagnosis and prognosis of GC.

## Materials and Methods

### Ethics Statement

This study was authorized by the human ethics committee of the Shanghai General Hospital affiliated of Shanghai Jiaotong University, People’s Republic of China (2017SQ018). Informed consent from these patients has been obtained before specimen collection.

All patients received primary tumor resection at Shanghai General Hospital and were diagnosed with GC based on histopathology after surgery. There was no preoperative radiotherapy or chemotherapy among these patients. 6 GC tissues with N0 (with no lymph node metastasis) and 6 GC tissues with N3 (with more than 7 lymph node metastasis) were used for miRNA microarray. 6 paired specimens were used for microarray analysis of lncRNAs and circRNAs. 60 paired tissues were used for validation by real-time PCR. Collected from 52 GC patients and 30 healthy people respectively in 2016-2017, peripheral blood were obtained before the operation and then the plasmas were isolated.

### Sample Collection and RNA Isolation

All the GC specimens including tissues and blood, were obtained from patients who received surgical resection for GC at Shanghai General Hospital affiliated of Shanghai Jiaotong University. Before RNA extraction, all specimens were snap-frozen instantly and stored at −80^◦^C. Blood samples (5 mL) were collected from all subjects in EDTA tubes and centrifuged at 3,000 rpm for 10 min at 4^◦^C, then the plasma was cautiously collected and also keep it at −80^◦^C until use. Total RNA extraction from tissues and plasma samples used TRIzol reagent (Invitrogen, Carlsbad, CA, USA) and TRNzol A+ (TIANGEN, Beijing, China). miRNA used miRcute Serum/plasma miRNA isolation kit (TIANGEN, Beijing, China) according to the manufacturer’s protocols. After adding denaturing solution (Ambion) for normalization of the sample-to-sample variation, 1 ul of synthetic external control (1 umol/L; TIANGENN) was spiked into each sample.

### MiRNA, lncRNA, and circRNA Microarray Expression Profiling

The laboratory of the OE Biotechnology Company (Shanghai, China) was in charge of the microarray profiling. NanoDrop ND-2000 (Thermo Scientific) was used for quantified analysis of total RNA. Agilent Bioanalyzer 2100 (Agilent Technologies) was used for the assessment of RNA integrity. Following the manufacturer’s standard protocols, the sample labeling, microarray hybridization and washing were performed. 6 GC tissues with N0, 6 GC tissues with N3 samples were transcribed to double-stranded cDNA, which was synthesized into the labeled cDNA and hybridized onto the Human miRNA array V4.0 (4×180 K, Agilent). Then t-test and p-value correction for False Discovery Rate (FDR) were applied to evaluating different expressions of miRNAs. 6 paired tissues samples were transcribed to double-stranded cDNA and hybridized onto the Human lncRNA array V4.0. Check the size and purity of the sample, and achieve the original data. To profile GC circRNA expression in the discovery cohort, total RNA was treated with RNase R for linear RNA removal and circRNA enrichment; then a random primer was in deployment and reverse-transcribed to fluorescence-labeled cRNA. Last, the fluorescent cRNAs were hybridized onto the Arraystar Human circRNA Array. The lncRNAs and circRNAs primary analysis of the raw data was concluded with Genespring software (Version 12.5, Agilent Technologies). Additionally, t-test and p-value correction for FDR were applied to evaluating different expressions of lncRNAs and circRNAs. The value of fold change was ≥2 and FDR p < 0.05 was statistically significant.

### Bioinformatics Analysis

To compare the noncoding RNAs and mRNA expression, we conducted Hierarchical Clustering in this study. Using the limma package in the Bioconductor package (http://www.bioconductor.org/), and R was used to run the instruction code. For each differentially expressed RNAs (DERs), the Pearson Correlation Coefficient (PCC) of its expression value with expression value of each mRNA were calculated. When the absolute value of PCC was <0. 8 and that of P-value was <0. 05, they were statistically relevant. Gene Ontology (GO) function and Kyoto Encyclopedia of Genes and Genomes (KEGG) pathway enrichment analyses were applied to predict the biological function of mRNAs. We performed GO annotations by using a DAVID online tool on the screened DERs. KEGG pathway analysis of DERs was performed using the KOBAS online analysis database (http://kobas.cbi.pku.edu.cn/). Furthermore, hypergeometric cumulative distribution function was performed to calculate the enrichment of functional terms in annotation of co-expressed mRNAs. Logically, the core transcription factors (TFs), which could trans-regulate the specific lncRNAs, involve certain biological pathways. It was predicted that differentially expressed lncRNAs possibly had participated in pathways regulated by TFs using Pearson correlation analyses and have calculated the correlation between TFs and lncRNAs. The TF-lncRNA-gene network was constructed by using hypergeometric cumulative distribution function of MATLAB 2012b and Cytoscape software (http://www.cytoscape.org). In addition, we constructed circRNA-miRNA network based on the binding capacity of circRNA on miRNA.

### Quantitative Reverse Transcription Polymerase Chain Reaction Analysis

The cDNAs were acquired by reverse transcription from total RNA with a PrimeScriptTM RT kit (Takara Bio Inc, Japan) and miRNA RT Enzyme Mix (TIANGEN, Beijing, China). The quantification of PCR product was evaluated by the level of fluorescence emitted by QuantiNova SYBR Green PCR Kit (Qiagen, Germany). GAPDH worked as an internal control for lncRNAs and circRNAs. U6 used as an internal control for miRNAs in tissues. Considering these synthetic miRNAs were exogenous references, which could not fully reflect the degradation degree of different samples. Thus, we added consensus external control as an internal reference in plasma. qRT-PCR primers from ShengGong (Shanghai, China) and RiboBio (Guangzhou, China) are listed in **Supplementary Table 1**. The qRT-PCR was conducted on LightCycler 480 RealTime PCR System (Roche Diagnostics) in 96-well plates at 95°C for 120 seconds, followed by 40 cycles of 95°C for 5 seconds, 60°C for 10 seconds, and then 70°C for 10 seconds. The relative levels of noncoding RNAs in tissue specimens and plasma were calculated using the comparative 2^-△△CT^ method, which was related to internal reference (GAPDH or U6) and endogenous reference.

### Statistical Analysis

Statistical analyses were performed by SPSS 21.0 software (IBM, Armonk, NY, USA). Fisher’s exact/chi-squared test and FDR were used for significance detection, where p-value denotes the significance of GO term and pathway correlated to the conditions. The smaller FDR indicates smaller error in judging the p-value. To compare two groups with normally distributed variables, we used student’s t-test in this investigation. For abnormally distributed variables, median and interquartile range (IQR) was used as the standard for comparisons. Student’s t-test and chi-square tests of variance were used to evaluate clinicopathological characteristics. The receiver operating characteristics (ROC) was applied to determine whether these ncRNAs had the capability for early diagnosis. Youden’s index was used to generate the optimal cut-off value for each ncRNA. In this study, survival analysis refers to the Overall Survival Kaplan-Meier Estimate.

## Result

### MiRNA Expression Profiles in GC Progression and Bioinformatics Prediction Analysis

**Figure 1:**
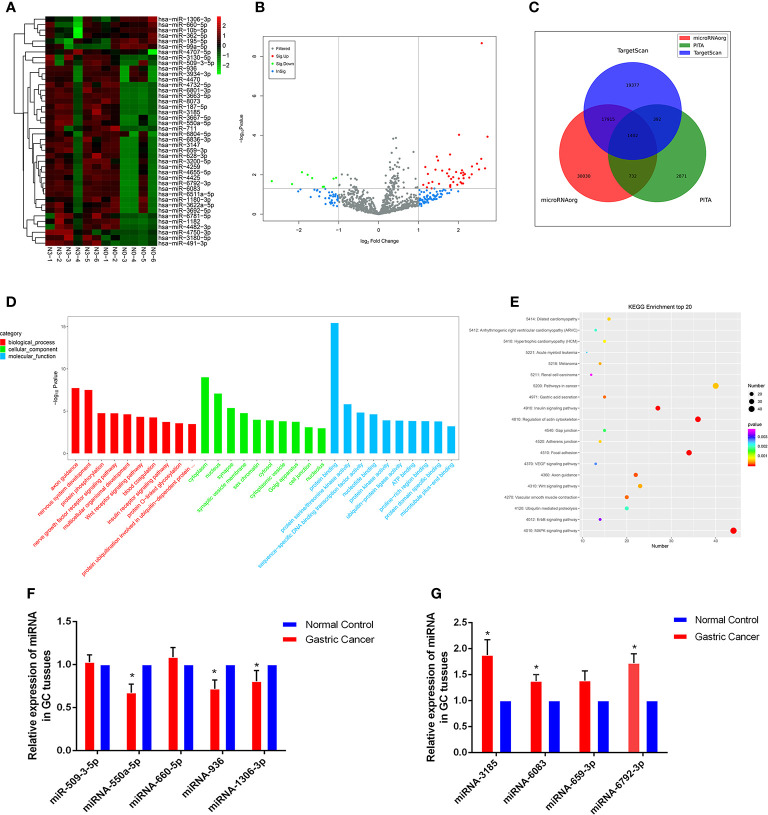
Profiling of miRNAs in the tissues from GC patients with N0 and N3 group and the expressions of miRNAs GC tissues. **(A)** Heat map shows the upregulated and down-regulated miRNAs in N0 *vs* N3 group. (N0 for no lymph node metastasis, and N3 for at least 7 lymph node metastasis). Each column represents the expression profile of a tissue sample, and each row corresponds to a miRNA. High expression level is indicated by “red” and lower levels by “blue”. **(B)** Volcano plot shows tp-regulated and down-regulated circRNAs in cancer *vs* normal group. Higher expression levels are indicated by “red”, lower expression levels are indicated by “green”, and no significant difference is indicated by other colors. **(C)** TargetScan, PITA, microRNAorg database for target gene prediction of differential miRNAs. **(D)** GO analysis of miRNAs in N0 *vs* N3 group. **(E)** KEGG analysis of miRNAs in N0 *vs* N3 group. **(F, G)** Expression of nine miRNAs in GC tissues compared to normal controls. *P < 0.05.

High-throughput sequencing was processed using tissue samples from 6 patients with N0 and the other 6 patients with N3 to assess miRNA expression profiles in GC progression. The primary data were showed in the manner of Heat maps and Volcano plot (**Figures 1A, B**). Among the 42 differentially expressed miRNAs, 36 were upregulated and 6 were downregulated in GC tissues relative to normal tissues Databases. Then, we predicted the potential targets gene by miRNA target prediction tools including TargetScan, PITA, and microRNAorg (**Figure 1C**). Overlapping the results of three prediction tools, we used them for subsequent GO and KEGG analysis. GO analysis was divided into three functional groups, including molecular function, biological processes, and cell composition. For the biological process, DNA-dependent transcriptional regulation were significantly enriched; For the cell composition, cytoplasm and nucleus were still dominant, and for the molecular function, protein binding was more abundant (**Figure 1D**). KEGG analysis results indicated that the MAPK signaling pathway and Pathways in cancer pathway were more abundant (**Figure 1E**), and these two pathways played an vital role in the occurrence and development of gastric carcinoma. Through GO and KEGG pathway analysis results, we selected 9 miRNAs (miR-509-3-5p, miR-550a-5p, miR-660-5p, miR-936, miR-1306-3p, miR-3185, miR-6083, miR-659-3p and miR-6792-3p) which were the most relevant to gastric cancer for subsequent validation.

### Validation of Selected miRNAs by qRT-PCR

We explored the clinical value of these miRNAs using 30 pairs of fresh gastric cancerous and paracancerous tissues. The results of qRT-PCR showed that 6 miRNAs were consistent with the chip results among the 9 miRNAs screened by GO analysis and KEGG analysis. The levels of miRNA-550a-5p, miRNA-936 and miRNA-1306-3p expression in gastric carcinoma were dramatically lower than those in pericarcinomatous tissue, while the levels of miRNA-3185, miRNA-6083 and miRNA-6792-3p expression were observably higher in GC tissues than NCs (**Figures 1F, G**). In-depth analysis of qRT-PCR data, it was found that miRNA-6792-3p expression level has obviously positive correlation with gastric cancer disease (TNM) stage and lymphatic metastasis (**Supplementary Figures 1A, B**). Meanwhile, miRNA-1306-3p expression level has negative correlation with gastric cancer disease (TNM) stage and lymphatic metastasis (**Supplementary Figures 1C, D**).

**Figure 2:**
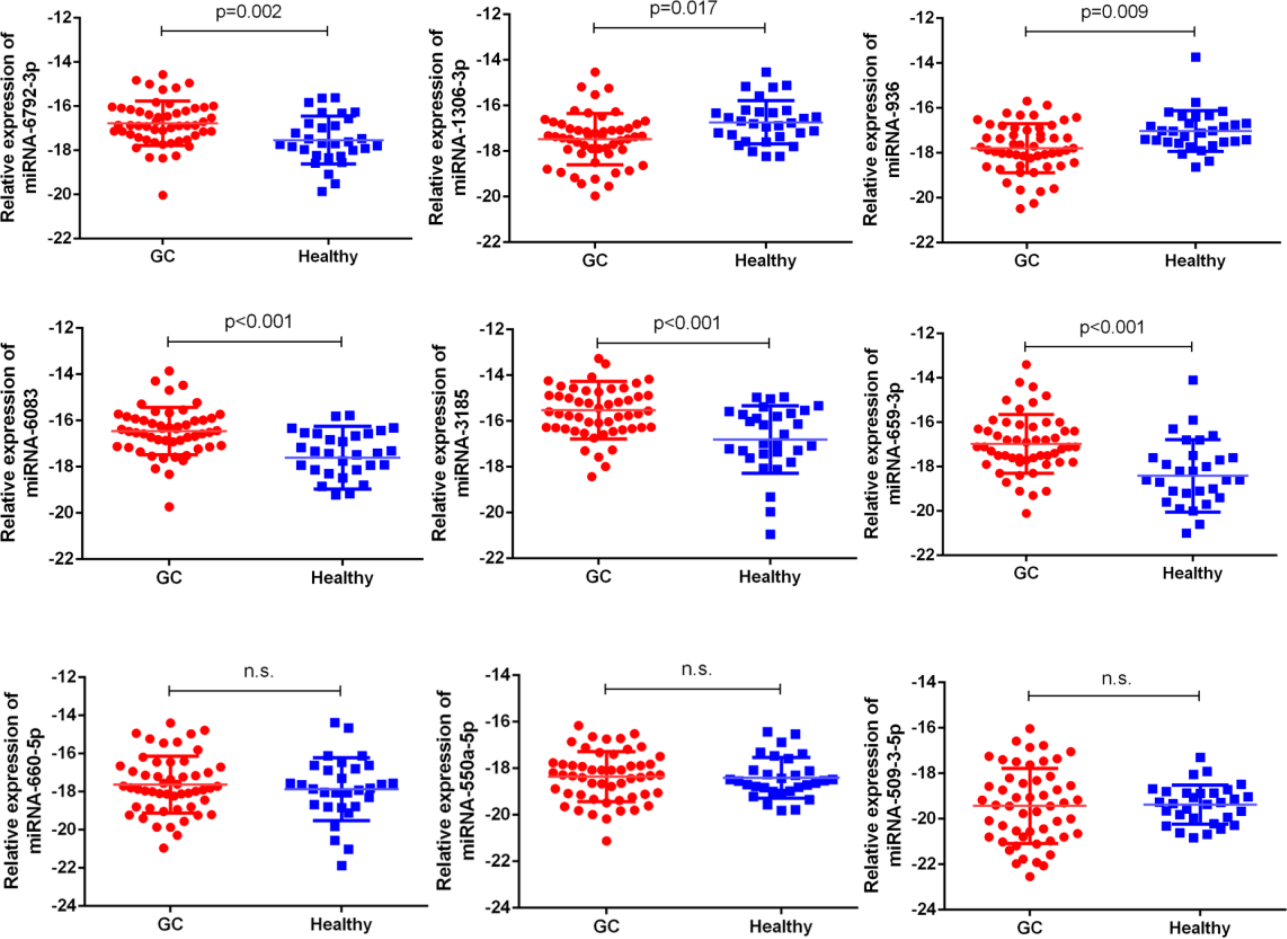
The expressions of nine miRNAs in GC bloods compared to healthy controls. The correlation of their -ΔCt value was determined. n.s., no significant.

Next, our team detected the expression levels of the nine miRNAs previous mentioned in the plasma of 52 patients with gastric cancer and 30 normal group (**Figure 2**). We found that the expression of miRNA-936, miRNA-1306-3p, miRNA-3185, miRNA-6083 and miRNA-6792-3p in plasma was consistent with that of GC tissues, in which the expression of miRNA-936 and miRNA-1306-3p were lower in circulating samples than that in NCs, and miRNA-3185, miRNA-6083, miRNA-659-3p and miRNA-6792-3p were highly expressed in plasma comparing the healthy group. From the depth analysis of qRT-PCR data and clinic pathological character of GC patients, it was found that the expression of miRNA-936, miRNA-1306-3p and miRNA-659-3p was linked with TNM stage and lymphatic metastasis, while miRNA-3185 was only related with TNM staged, miRNA-6792-3p was positively correlated lymphatic metastasis in patients. In addition, the expression of miRNA-1306-3p, miRNA-659-3p and miRNA-6083 were closely connected with vascular invasion of GC patients (**Table 1**). Furthermore, we explored the relationship between these miRNAs and traditional tumor markers including CA724, CA199 and CEA, and we found miRNA-6083 was positively correlated with CEA, and miRNA-6792-3p was positively correlated with CA724 (**Supplementary Figures 1G, H**). These data suggest that these miRNAs can serve as a cancer biomarker in GC.

**Table 1:** Correlation between clinicopathological features and expression levels of miRNA in plasma with gastric adenocarcinoma.

Characteristic	Case	miR-936		miR-1306		miR-659-3P	
		Median (range), △CT	p-vaule	Median (range), △CT	p-vaule	Median (range), △CT	p-vaule
Gender							
Male	36	17.81 (15.87-20.48)	0.677	17.33 (15.18-19.54)	0.439	17.07 (14.24-20.10)	0.500
female	16	17.90 (15.69-19.65)		17.50 (14.53-19.96)		16.96 (13.39-19.27)	
Age (years)							
≤65	26	17.84 (16.22-20.48)	0.487	17.57 (15.52-19.96)	0.370	17.09 (13.39-20.10)	0.819
>65	26	17.81 (15.69-19.65)		17.32 (14.53-19.43)		17.07 (14.35-18.34)	
Tumor size (cm)							
<5	25	17.61 (15.87-19.72)	0.318	17.29 (15.18-19.96)	0.447	17.06 (13.39-20.10)	0.949
≥5	27	17.94 (15.69-20.48)		17.42 (14.53-19.43)		17.08 (14.24-19.10)	
Differentiation							
Moderate/ well	18	17.87 (15.56-20.48)	0.887	17.70 (14.53-19.96)	0.393	17.10 (14.35-20.10)	0.375
Poor	29	18.02 (16.31-19.59)		17.31 (16.22-19.54)		16.93 (13.39-18.34)	
TNM stage							
I, II	20	17.35 (15.69-19.72)	**0.049**	17.22 (14.53-18.50)	**0.042**	16.38 (13.39-20.10)	**0.022**
III, IV	32	17.99 (15.87-20.48)		17.45 (15.18-19.96)		17.42 (14.24-19.27)	
Lymph node metastasis							
Negative	14	17.26 (15.69-18.08)	**0.004**	17.01 (14.53-17.89)	**0.004**	16.07 (13.39-18.31)	**0.002**
Positive	38	17.99 (15.87-20.48)		17.59 (15.18-19.96)		17.42 (14.24-20.10)	
Nerve invasion							
Yes	29	17.88 (15.87-20.48)	0.543	17.70 (15.18-19.96)	0.072	17.39 (14.24-19.27)	0.085
No	23	17.64 (15.69-19.72)		17.32 (14.53-19.54)		16.76 (13.39-20.10)	
Vessel invasion							
Yes	34	17.91 (15.87-20.25)	0.538	17.59 (15.18-19.96)	**0.023**	17.46 (14.24-20.10)	**0.001**
No	18	17.69 (15.69-20.48)		17.08 (14.53-19.16)		16.31 (13.39-17.60)	
Characteristic	Case	miR-3185		miR-6083		miR-6792-3P
		Median (range), △CT	p-vaule	Median (range), △CT	p-vaule	Median (range), △CT	p-vaule
Gender							
Male	36	15.19 (10.92-18.43)	0.178	16.37 (13.85-19.73)	0.929	16.89 (14.56-20.03)	0.774
female	16	15.82 (14.17-17.26)		16.49 (14.29-18.08)		16.78 (15.00-17.89)	
Age (years)							
≤65	26	16.04 (10.92-18.43)	0.018	16.37 (14.47-19.73)	0.528	16.89 (14.56-20.03)	0.784
>65	26	15.13 (13.26-16.59)		16.55 (13.85-17.73)		16.78 (14.68-18.35)	
Tumor size (cm)							
<5	25	16.05 (13.26-18.43)	0.058	16.35 (13.85-19.73)	0.798	16.89 (14.56-20.03)	0.776
≥5	27	15.27 (10.92-17.57)		16.51 (14.29-18.32)		16.86 (14.68-18.35)	
Differentiation							
Moderate/well	18	15.60 (14.07-18.43)	0.710	16.38 (13.85-19.73)	0.810	16.49 (14.56-20.03)	0.504
Poor	29	15.93 (10.92-17.57)		16.41 (14.69-17.73)		17.02 (15.00-18.35)	
TNM stage							
I, II	20	16.28 (13.50-17.99)	**0.016**	16.12 (13.85-19.73)	**0.011**	16.86 (14.56-20.03)	0.296
III, IV	32	15.30 (10.92-18.43)		16.72 (14.47-18.32)		16.89 (14.68-18.35)	
Lymph node metastasis							
Negative	14	16.05 (13.50-17.57)	0.208	15.85 (13.85-17.23)	**0.006**	16.44 (14.56-17.52)	**0.041**
Positive	38	15.57 (10.92-18.43)		16.63 (14.47-19.73)		16.89 (14.68-16.89)	
Nerve invasion							
Yes	29	15.16 (10.92-18.43)	0.030	16.53 (13.85-18.32)	0.392	16.86 (14.68-18.35)	0.768
No	23	16.26 (13.50-17.99)		16.34 (14.29-19.73)		16.88 (14.56-20.03)	
Vessel invasion							
Yes	34	15.51 (10.92-18.43)	0.736	16.63 (13.85-19.73)	**0.003**	16.89 (15.16-20.03)	0.091
No	18	15.73 (13.50-16.74)		15.85 (14.29-17.64)		16.67 (14.56-18.25)	

The bold values highlight statistical significance.

In addition, we explored the expression levels of the six miRNAs expression in portal vein serum and thirty matched peripheral serum of 30 patients with GC to identify the comparison of miRNAs expression between the portal and peripheral serum. We speculated that circulating miRNAs released from portal venous blood might present a higher expression level than those in peripheral venous blood. But all the six miRNAs showed no difference of expression levels in the portal and peripheral serum totally, the results were not statistically significant (**Supplementary Figure 2**).

### LncRNA Expression Profiles and Bioinformatics Data Analysis

**Figure 3:**
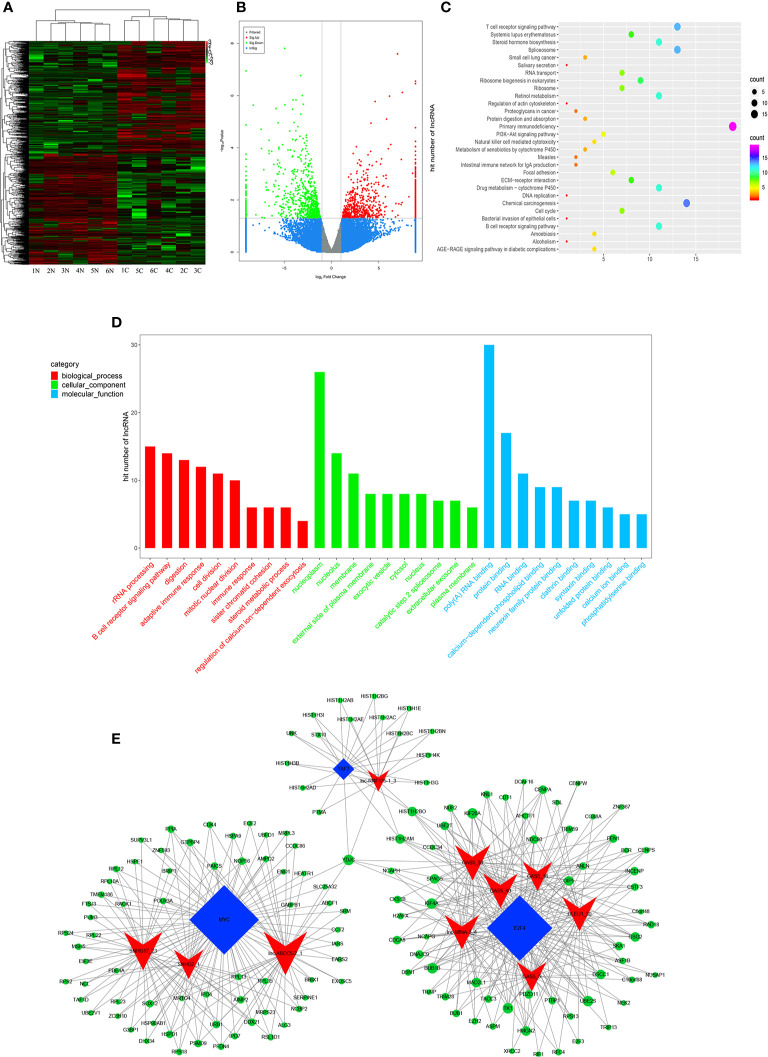
Profiling of lncRNAs in GC tissues compared to adjacent mucosa. **(A)** Heat map shows the upregulated and down-regulated lncRNAs in cancer *vs* normal group. (C for cancer, and N for normal mucosa). Each column represents the expression profile of a tissue sample, and each row corresponds to a lncRNA. High expression level is indicated by “red” and lower levels by “green”. **(B)** Volcano plot shows tp-regulated and down-regulated circRNAs in cancer *vs* normal group. Higher expression levels are indicated by “red”, lower expression levels are indicated by “green”, and no significant difference is indicated by other colors. **(C)** KEGG analysis of lncRNAs in cancer *vs* normal group. **(D)** GO analysis of lncRNAs in cancer *vs* normal group. **(E)** TF-lncRNA-targetgene network diagram.

High-throughput lncRNA microarray was performed using 6 GC tissues compared with non-cancerous matched tissues to determine lncRNA expression profiles in gastric cancerous progression. The data were revealed in the way of Heat maps and Volcano plots (**Figures 3A, B**), 760 remarkable upregulated and 739 downregulated lncRNAs were detected in GC tissues (>2-fold change; P-value<0. 05). KEGG and GO pathway annotations were adopted to predict the functions of the top 200 abnormal expressed lncRNAs. GO biological process displayed that the most relevant biological process of differentially expressed lncRNAs were rRNA processing, B cell receptor signaling pathway, and digestion (**Figure 3D**). GO cellular component showed that nucleoplasm, nucleus and membrane were enriched. GO molecular function analysis suggested that some functional pathways involved in the pathogenesis were enriched, such as poly(A) RNA binding, protein binding, RNA binding and so on. KEGG pathway analysis indicated that 38 pathways corresponded to these lncRNAs and the most enriched network were primary immunodeficiency, composed of 19 lncRNAs (**Figure 3C**). Among these pathways, the lncRNA category ‘Chemical carcinogenesis’ and ‘Spliceosome’, were possibly involved in the carcinogenesis of GC. The lncRNA category “PI3K-Akt” signaling pathway, is involved in proliferation migration and angiogenesis of GC. The correlation analysis of lncRNAs and mRNAs showed that the differentially expressed lncRNAs are closely associated with lots of mRNAs.

Many studies have reported that some lncRNAs involved in a few biological pathways might have associated with TFs (24, 25). The top 200 lncRNAs were summarized to predict the possibly relevant TFs using Pearson correlation analyses and to calculate the correlation between TFs and lncRNAs, in which the most frequently predicted TFs were E2F4, MYC, EBF1, TAF1 and TAF7 (**Supplementary Figure 3**). To further explore the trans-regulating functions of lncRNAs, we constructed a core TF-lncRNA-target-gene network. The network contained 155 network nodes including 3 core TFs (E2F4, MYC and TAF7), 10 lncRNAs with aberrant expression and 142 target genes (**Figure 3E**). The co-expression network may suggest that the interregulation of lncRNAs and mRNAs is involved in GC.

### Evaluation of Candidate lncRNAs by qRT-PCR

**Figure 4:**
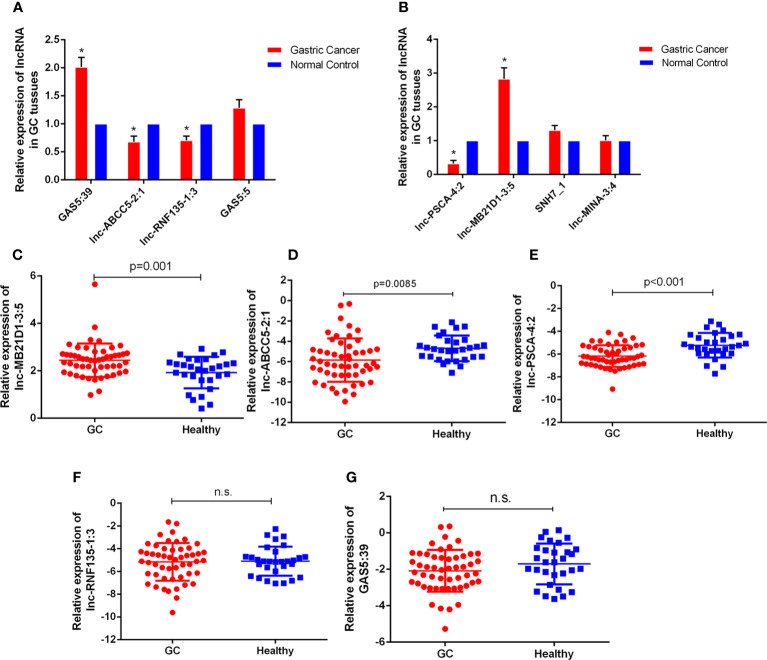
The expressions of lncRNAs GC tissues and bloods. **(A, B)** Expression of eight lncRNAs in GC tissues compared to normal controls. The correlation of their -ΔCt value was determined. *P < 0.05 **(C–G)** Expression of five lncRNAs in GC bloods compared to healthy controls. n.s., no significant.

With the lncRNA microarray results, we selected the lowest and highest abnormal expression and other critical lncRNAs in the core lncRNA-target-gene network for verification. qRT-PCR results showed that 5 lncRNAs were abnormally expressed in 30 pairs of fresh gastric carcinoma and matched adjacent tumor tissues (**Figures 4A, B**), which were consistent with bioinformatics analysis. GAS5:39 and lnc-MB21D1-3:5 were high expressed in the identified gastric cancer tissues than NCs. The expression of lnc-ABCC5-2:1, lnc-PSCA-4:2 and lnc-RNF135-1:3 in gastric cancer tissues was significantly lower than pericarcinomatous tissue. From an in-depth analysis of qRT-PCR data, it was found that lnc-PSCA-4:2 was negatively correlated with tumor differentiation, the higher level of lnc-PSCA-4:2 expression, the lower the degree of differentiation. The expression level of lnc-MB21D1-3:5 was positively correlated with the clinical stage (**Supplementary Figures 1E, F**). The above results demonstrated that these screened lncRNAs were correlated with stage and tumor differentiation.

Furthermore, we detected the expression of these five lncRNAs selected by microarray data in the plasma of 52 GC patients and 30 healthy people. qRT-PCR results showed that the expression of lnc-MB21D1-3:5, lnc-PSCA-4:2 and lnc-ABCC5-2:1 in plasma was consistent with GC tissues. The expression of lnc-PSCA-4:2 and lnc-ABCC5-2:1 in the plasma of gastric cancer patients was lower than healthy control, and lnc-MB21D1-3:5 was up-regulated in the plasma samples compared with healthy control (**Figures 4C–G**). Through the depth analysis of qRT-PCR data and clinicopathological features of GC patients it was found that the expression of lnc-PSCA-4:2 in the plasma was closely connected to the disease(TNM)stage, vascular invasion and lymphatic metastasis of GC patients, and lnc-MB21D1-3:5 was significantly associated with the degree of differentiation of gastric cancer (**Table 2**). Notably, lncRNAs can be detected in plasma and hold great promise as biomarkers.

**Table 2:** Correlation between clinicopathological features and expression levels of lncRNA in plasma with gastric adenocarcinoma.

Characteristic	Case	lnc-MB21D1-3:5		lnc-PSCA-4:2		lnc-ABCC5-2:1	
		Median (range), △CT	p-vaule	Median (range), △CT	p-vaule	Median (range), △CT	p-vaule
Gender							
Male	36	2.62 (0.98-3.84)	0.488	6.36 (4.27-7.35)	0.751	6.07 (9.32-0.23)	0.960
female	16	2.36 (1.62-5.56)		6.35 (4.10-9.06)		6.45 (0.47-9.93)	
Age (years)							
≤65	26	2.43 (0.98-2.84)	0.840	6.52 (4.10-7.49)	0.227	6.44 (0.32-9.63)	0.370
>65	26	2.45 (1.14-5.65)		6.05 (4.27-9.06)		5.86 (0.47-9.93)	
Tumor size (cm)							
<5	25	2.59 (0.98-5.65)	0.985	6.36 (4.58-9.06)	0.356	6.45 (0.32-8.87)	0.679
≥5	27	2.36 (1.14-3.62)		6.23 (4.58-9.06)		5.74 (0.47-9.93)	
Differentiation							
Moderate/well	18	2.20 (1.14-3.62)	**0.018**	6.42 (4.10-9.06)	0.768	6.07 (1.74-9.93)	0.904
Poor	29	2.63 (0.98-5.62)		6.34 (4.58-7.49)		6.38 (0.32-9.23)	
TNM stage							
I, II	20	2.60 (0.98-5.65)	0.463	6.77 (4.29-9.06)	**0.025**	5.69 (0.32-8.87)	0.259
III, IV	32	2.36 (1.14-3.31)		6.13 (4.10-7.14)		6.27 (1.74-9.93)	
Lymph node metastasis							
Negative	14	2.66 (0.98-5.65)	**0.004**	6.88 (5.43-9.06)	**0.025**	5.12 (0.32-8.05)	0.259
Positive	38	2.36 (1.14-3.31)		6.13 (4.10-7.27)		6.38 (1.74-9.93)	
Nerve invasion							
Yes	29	2.44 (1.44-5.65)	0.706	6.22 (4.10-7.14)	0.077	6.38 (3.12-9.93)	0.176
No	23	2.46 (0.98-3.84)		6.55 (4.27-9.06)		5.14 (0.32-8.87)	
Vessel invasion							
Yes	34	2.47 (1.14-5.655)	0.729	6.13 (4.10-7.49)	**0.036**	6.31 (1.74-9.93)	0.281
No	18	2.45 (0.98-3.84)		6.60 (4.27-9.06)		5.12 (0.32-9.23)	

The bold values highlight statistical significance.

### Diagnostic Value of the Candidate miRNAs and lncRNAs

**Figure 5:**
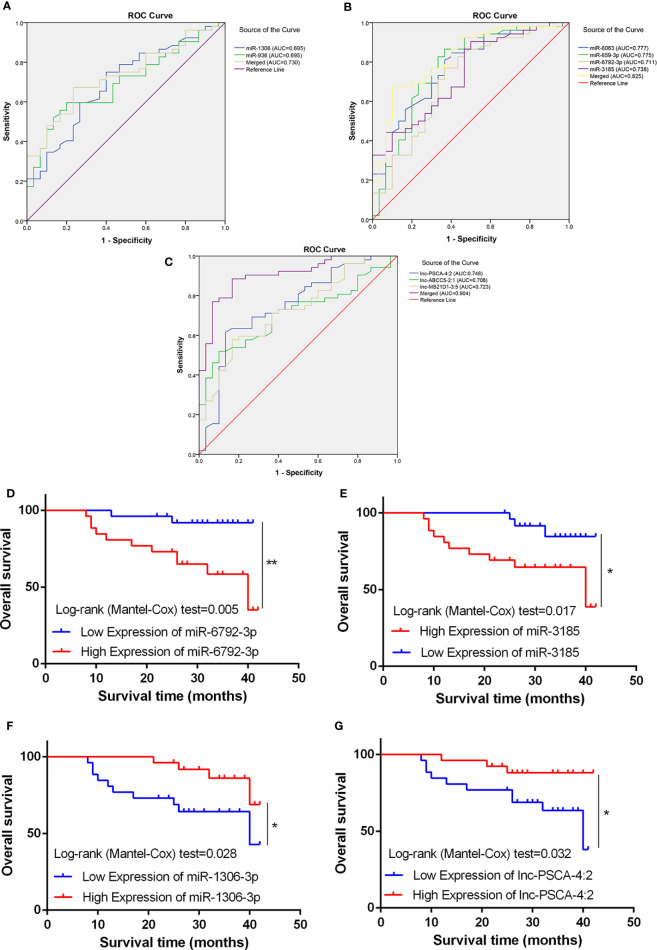
Diagnostic potential of miRNA and lncRNA. **(A)** The area under the ROC curve (AUC) value of miRNA-936 and miRNA-1306-3p was 0.675 and 0.658 respectively. **(B)** The AUC value of miRNA-3185, miRNA-6083, miRNA-659-3p and miRNA-6792-3p was 0.775, 0.739, 0.777 and 0.711 respectively. **(C)** The AUC value of lnc-PSCA-4:2, lnc-ABCC5-2:1 and lnc-MB21D1-3:5 was 0.746, 0.708, and 0.723 respectively. **(D, E)** Kaplan-Meier overall survival curve revealed patients with higher miR-6792-3p and miR-3185 expression showed a reduced survival time. **(F, G)** Kaplan-Meier overall survival curve revealed patients with lower miR-1306-3p and lnc-PSCA-4:2 expression showed a reduced survival time. *p < 0.05, **p < 0.01.

Using the receiver operating curve (ROC) analysis, we explored the possibility of these differentially expressed ncRNAs as molecular markers in the blood of GC patients. The AUCs were 0.675, 0.658, 0.775, 0.739, 0.777 and 0.711 for miRNA-936, miRNA-1306-3p, miRNA-3185, miRNA-6083, miRNA-659-3p and miRNA-6792-3p, respectively (**Figures 5A, B**). Furthermore, when the two low and four high expression of miRNAs were combined as a panel separately and the AUCs were 0.730 (95% CI, 0.623-0.838) and 0.825 (95% CI, 0.732-0.918) respectively, it showed a higher accuracy than an individual miRNA in discriminating between GC patients and healthy controls. Then we used ROC analysis to discover the possibility of these aberrant expressed lncRNAs as molecular markers. The AUCs were 0.746, 0.708 and 0.723 for lnc-MB21D1-3:5, lnc-PSCA-4:2 and lnc-ABCC5-2:1, respectively (**Figure 5C**). As expected, when the three lncRNAs were merged as a panel, it demonstrated a higher sensitivity and specificity than any individual lncRNA to discriminate gastric cancer patients from healthy controls (AUC:0.904; 95% CI, 0.838–0.970). Additionally, Kaplan-Meier overall survival curve revealed that patients with higher miR-6792-3p and miR-3185 expression showed a reduced survival time (**Figures 5D, E**). Patients who had low levels of miR-1306-3p and lnc-PSCA-4:2 in circulating of GC patients had significantly shorter overall survival rate (**Figures 5F, G**).

Taken together, these findings suggested miRNA-936, miRNA-1306-3p, miRNA-3185, miRNA-6083, miRNA-659-3p, miRNA-6792-3p, lnc-MB21D1-3:5, lnc-PSCA-4:2 and lnc-ABCC5-2:1 were a suitable circulating biomarker in diagnosing GC.

### CircRNAs Expression Profiles in GC and Bioinformatics Prediction With Clinical Implication

**Figure 6:**
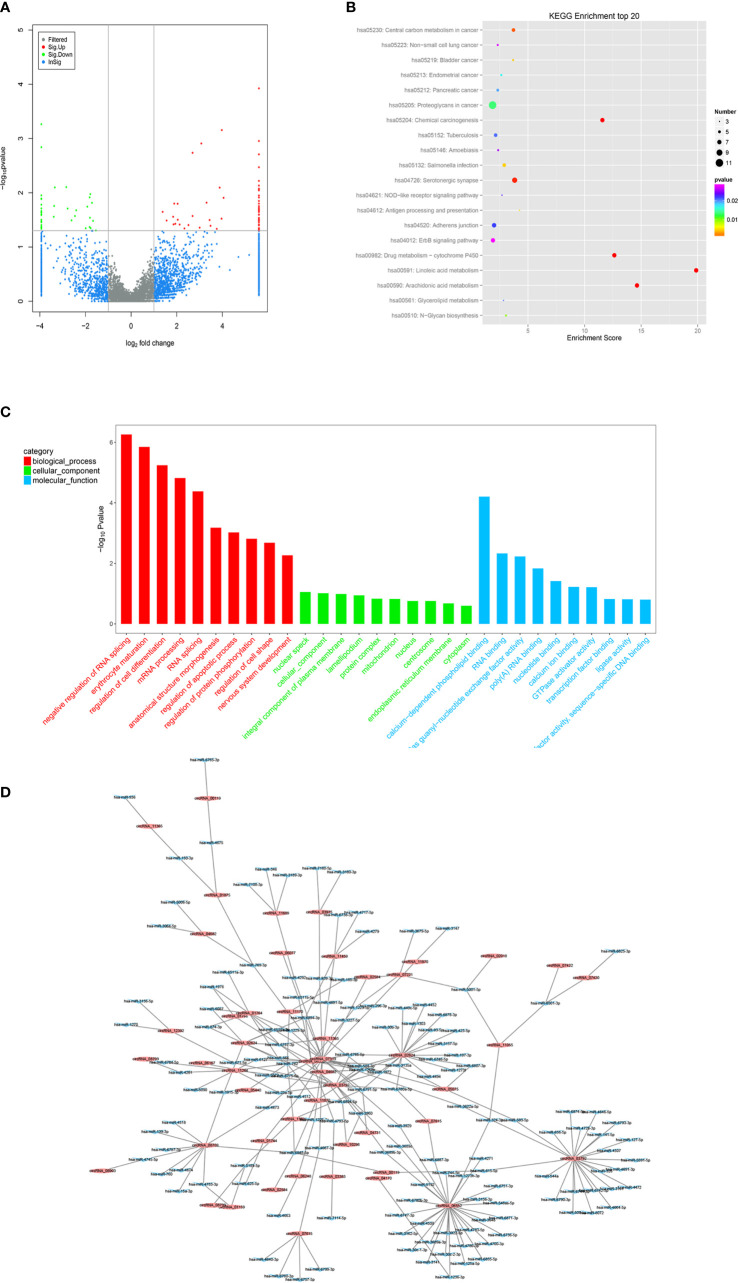
Profiling of circRNAs in GC tissues compared to adjacent mucosa. **(A)** Volcano plot shows tp-regulated and down-regulated circRNAs in cancer vs normal group. Higher expression levels are indicated by “red”, lower expression levels are indicated by “green”, and no significant difference is indicated by other colors. **(B)** KEGG analysis of circRNAs in cancer vs normal group. **(C)** GO analysis of lncRNAs in cancer vs normal group. **(D)** circRNA-miRNA target interaction network diagram.

To determine the expression profiles of circRNAs in GC progression, we conducted high-throughput human circRNA microarray. Tissue samples from 6 GC tissues and 6 normal controls were used in this study. The data were displayed in the format of H Volcano plots (**Figure 6A**). Based on genomic origin, there are five types of circRNAs, which include exon circRNA, intron circRNA, antisense circRNA, sense overlapping circRNA and intergenic circRNA, we found that the primary kind of circRNAs was sense overlapping in our experiment. Bioinformatics prediction analysis suggested that these aberrant expressed circRNAs are connected with several significant biological processes, molecular functions, cellular constituent, and crucial signaling pathways (**Figures 6B, C**). Dysregulated circRNAs were significantly enriched in nuclear speck and lamellipodium of cellular component, negative regulation of RNA splicing and erythrocyte maturation in biological process, calcium-dependent phospholipid binding and RNA binding in molecular function. Those circRNAs were significantly enriched in several KEGG signaling pathways. Proteoglycan in cancer, chemical carcinogenesis, adherens junction and ErbB signaling pathway were the top pathways associated with GC. In order to reveal the co-expression pattern of circRNA-miRNA, the circRNA-miRNA co-expression networks were constructed based on the high-throughput RNA sequencing results and bioinformatics analysis (**Figure 6D**).

**Figure 7:**
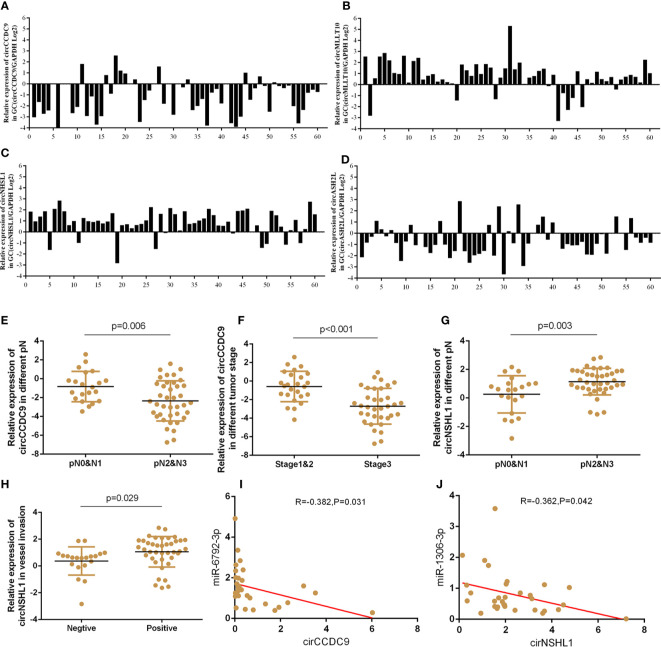
The expressions of circRNAs GC tissues and clinical information analysis. **(A–D)** Expression of circASHL2, circCCDC9, circNHSL1 and cirMLLT10 in GC tissues compared to normal controls. **(E, F)** The expression of circCCDC9 was negatively associated with TNM stage and lymphatic metastasis. **(G, H)** The expression of circNHSL1 was positively associated with vessel invasion and lymphatic metastasis. **(I)** The expression of circNHSL1 was negatively associated with that of miR-1306-3p. **(J)** The expression of circCCDC9 was negatively associated with that of miR6792-3p.

On the basis of circRNA bioinformatics predictions, we selected 4 circRNAs for further validation of the microarray consistency using qPCR. Since it could not be detected in blood, we increased the number of tissue samples to detect the expression of circRNAs. Results showed that circASHL2 and circCCDC9 were observably low expressed in tumor tissues, circNHSL1 and cirMLLT10 were markedly increased in tumor tissues compared pair-matched non-cancer adjacent tissues (**Figures 7A–D**). Clinic pathological character showed that the down-regulated circCCDC9 was negatively associated with TNM stage and lymphatic metastasis, the up-regulated circNHSL1 was positively associated with lymphatic metastasis and vascular invasion (**Figures 7E–H**). Furthermore, it was found that circCCDC9 was negatively correlated with the expression level of miRNA-6792-3p, and circNHSL1 was negatively correlated with miRNA-1306-3p (**Figures 7I, J**). There was potential binding site on circCCDC9 and circNHSL1 respectively for those miRNAs which were detected before. We will further detect the expression of circRNAs in plasma sample.

## Discussion

In spite of significant progress in cancer early diagnosis and therapy, the survival rate of patients with GC has not achieved satisfactory improvement over the past few decades. So far, there are several challenges in the diagnosis and treatment of gastric cancer. First, there is difficulty in detecting early diagnosis. The pathological and biological characteristics of gastric cancer for early symptoms lack specificity, and the majority of GC cases are diagnosed in their advanced stage (26). Second, the heterogeneity of gastric cancer makes that it was difficult to cure. The whole-genome analysis of gastric cancer shows that 10 core signaling pathways have genetic changes. The change of multigene and various pathways increase the difficulty of achieving effective treatments which may be leading to poor prognoses (27, 28). Therefore, there is an urgent need to develop novel, highly sensitive and specific molecular markers for detection and diagnosis of GC to improve patient outcomes.

MiRNA was detected more than 20 years ago, which had not been attached much importance until 2001. Many human miRNAs were identified, and had been put into use for early detection, which was aberrantly expressed frequently in tumor tissues due to the relationship with tumor growth, invasion, and metastasis (29–31). However, the miRNA in cancer tissues is not ideal for invasive procedure involved obtaining tissue samples. It was not until 2008 that an investigation discovered miRNA in the plasma of tumor sufferer might be serve as biomarkers for malignancy and they might be detected in other body fluids of cancer patients successively (32). In contrast to intracellular miRNA, humoral miRNAs were harmless biomarkers for early cancer detection, including GC. For example, Tsujiura et al. (33) found that five circulating miRNAs (miR-17-5p, miR-21, miR-106a, miR-106b and let-7a) were verified to be differently expressed in GC tissues and also changed circulating samples from both of pre-operative GC patients and healthy people. Contrary to the previous outline researches, Ren C et al. (34) demonstrated that the expression of miR-16 and miR-451 was associated with survival rate in GC patients, and high expression of miR-16 and miR-451 displayed better survival rate. They showed miR-16 and miR-451 might be applied to predict the prognosis and provided a new treatment target for GC patients. In addition, single miRNA in plasma of GC patients was not optimal for diagnosis on account of heterogeneity of tumor. Therefore, multiple merged miRNAs might enhance their diagnostic value for GC effectively. Although several estimated biomarkers for gastric cancer have been investigated, no single blood-based biomarker with satisfactory sensitivity or specificity has been introduced (35). In our study, we used N0 and N3 GC tissues to pick out dysregulated miRNAs. As we know, lymph node metastasis is one of the most important features of malignant tumors, including GC. On the other hand, the miRNA that associated with lymph node metastasis must be serve as oncogene or tumor suppressor gene. More importantly, we have never used this part of miRNA sequencing and bioinformatics data. In our research, we selected six miRNAs with microarray profiling followed by qRT-PCR validation. miRNA-3185, miRNA- 6083, miRNA-659-3p and miRNA-6792-3p were confirmed to be significantly upregulated in GC plasma, whereas miRNA-936 and miRNA-1306-3p were significantly downregulated in GC plasma. More importantly, the expression of these miRNAs is closely related to the clinical characteristics of GC patients. Surprisingly, miRNA-6083 was positively correlated with CEA, and miRNA-6792-3p was positively correlated with CA724 in the patient’s blood of GC. The diagnostic value of the six miRNAs was verified in GC plasma, and the encouraging results increasingly demonstrated the important roles for the six miRNAs in tumorigenesis and progression.

High-throughput sequencing has revealed that lncRNAs could be new regulators of cancer progression. Recently, it was found that circulating lncRNAs had the value of the detection of various cancer types. The reason why they became biomarkers was not only because specimen covered circulating lncRNA could be easily and noninvasively gained from cancer patients but also because these lncRNAs had high stability in body fluids (36, 37). In a recent study, Zhang K et al. (38) designed a research study to explore the possibility of lncRNA as a marker in GC. They constructed two biomarker panels, containing lncRNA-based Index I and CEA-based Index II based on logistic regression, to compared the diagnostic performance of five lncRNAs. The result showed that the Index I surpassed the Index II in GC patients and healthy controls with an AUC value of 0.90 (95% CI: 0.86-0.95). Interestingly, in contrast to preoperative plasma samples from GC patients, the AUC value of index I reduced notably by postoperative day 14, which indicated the panel of five lncRNAs could monitor tumor dynamics. The five-lncRNAs panel demonstrated a high diagnostic precision for GC detection. Esfandia F et al. (39) explored expression of a panel of lncRNAs including HULC, OIP5-AS1 and THRIL in 30 GC tissues and paired adjacent non-cancerous tissues, and ROC curve analysis showed diagnostic power of 0.72, 0.69 and 0.68 for THRIL, HULC and OIP5-AS1, respectively. The AUC value for combination of three lncRNAs was higher than that of HULC and OIP5-AS1, but did not lead to significant improvement of the diagnostic power. In our study, we found that lnc-ABCC5-2:1 were less expressed in circulating and tissue samples than normal controls, whereas lnc-MB21D1-3:5 and lnc-PSCA-4:2 were higher expressed in circulating and tissue samples than normal controls. Among them, the expression of lnc-PSCA-4:2 in blood is closely related to stage, vascular invasion and lymphatic metastasis of gastric cancer patients, and lnc-MB21D1-3:5 was significantly correlated with the differentiation degree of gastric cancer. Besides, the three identified lncRNAs all have not been studied in tumors. Moreover, both circulating lnc-MB21D1-3:5, lnc-ABCC5-2:1 and lnc-PSCA-4:2 were discovered for the first time to be valuable biomarkers of GC in our study. ROC curve analysis of the 3 lncRNAs showed that the AUC values of lnc-MB21D1-3:5, lnc-PSCA-4:2 and lnc-ABCC5-2:1 were 0.723, 0.708 and 0.746, respectively, while the combined diagnosis of 3 lncRNAs reached 0.902, which suggested that they could be function as promising biomarkers for gastric cancer.

CircRNA, an emerging member of ncRNAs, which originates from exons, introns or both and function as sponging miRNAs, is to regulate RNA transcription sponging proteins, to interact with proteins, and to translate proteins (40, 41). Nowadays, more than 100,000 types of circRNAs are discovered from different species, and the quantity of intracellular circRNA are ten-fold more than that of homogenetic linear isomer RNA in humans (42). Currently, numerous GC studies were concentrated on the expression level of circRNAs in GC tissue, while most of them were existed stably in plasma with an O-shaped closed structure and were resistant to exonuclease and RNases (43). However, several studies have already been proven the feasibility of circulating circRNAs for detection of GC. Chen S et al. (44) studied the expression of several circRNAs in blood and showed that hsa_circ_0000190 has a low expression level in gastric cancer plasma specimens. In particular, the expression of hsa_circ_0000190 was associated significantly with tumor diameter, lymph nodal metastasis, distal metastasis, tumor stage, and CA19-9 levels. Hsa_circ_0000190 had potential diagnostic performance for GC, with AUC of 0.775, specificity of 71.2% and sensitivity of 75.0%. Another study screened plasma samples of circRNAs expression profiles from 10 GC patients and 5 healthy individuals by using the microarray technique; and demonstrated that the expression of circ-KIAA1244 had decreased altogether in GC tissues, plasmas, and cells. Moreover, the low plasma level of circ-KIAA1244 had a significant relationship with tumor stage, lymph nodal metastasis and survival rate (45). Our study screened circRNAs expression profiles from 6 pair GC and paracancer tissues by using the microarray technique, and demonstrated that the expression of four circRNAs (circASHL2, circCCDC9, circNHSL1 and cirMLLT10) had dysregulated altogether in GC tissues. Moreover, the level of circCCDC9 and circNHSL1 had a significant relationship with tumor stage, lymph nodal metastasis and vessel invasion. Even though circRNAs could be resistant to exonuclease and RNases with an O-shaped closed structure, not every circRNA can be detected in the plasma. In our research, we tried to use different detection methods and these circRNAs still cannot be detected in plasma.

As we known, the entry of tumor cells and their secretory products into the portal system is a crucial step in the metastasis of the digestive tract. It was speculated that circulating ncRNAs released from portal venous blood might present a higher expression level than those in peripheral venous blood. Although no significant differences, our result might testify the hypothesis to some extent. Further researches containing larger samples are warranted.

Our study has some limitations. Firstly, due to time and economic problems, the sample size was small. More substantial and more diverse samples should be taken into consideration and further exploration. Secondly, it was predicted that ncRNA had functioned indirectly with GO and KEGG pathway analyses of relevant mRNA based on the results of microarray data, with unclear the roles of the ncRNAs in GC pathogenesis. We need to perform further functional experiments of these ncRNAs which we had validated on gastric cancer cell lines and xenograft models to further demonstrate their roles in GC prognosis.

## Conclusion

Taken together, we identified that several ncRNAs(miRNA-936, miRNA-1306-3p, miRNA-3185, miRNA- 6083, miRNA-659-3p, miRNA-6792-3p, lnc-ABCC5-2:1, lnc-MB21D1-3:5, lnc-PSCA-4:2, circASHL2, circCCDC9, circNHSL1 and cirMLLT10) could be useful to distinguish GC patients and also to predict the prognosis and prognosis of GC patients. Further studies on a larger cohort of patients are needed to validate our findings. More prospective research are needed to explore the function of circulating ncRNAs as reliable and effective biomarkers in gastric carcinoma diagnosis and prognosis.

## Data Availability Statement

The datasets presented in this study can be found in online repositories. The names of the repository/repositories and accession number(s) can be found below: GEO and accession GSE173215 and GSE174237.

## Ethics Statement

The studies involving human participants were reviewed and approved by Shanghai General Hospital ethics committee. The patients/participants provided their written informed consent to participate in this study. Written informed consent was obtained from the individual(s) for the publication of any potentially identifiable images or data included in this article.

## Author Contributions

ZY wrote the main manuscript and analyzed the data. ZY, JS and ZR performed the experiments. ZY and CH designed the study. Final manuscript were reviewed and approved by all authors without disagreement.

## Funding

This work was supported by the National Natural Science Foundation of China (Grant No. 817725276), Shanghai Municipal Education Commission-Gaofeng Clinical Medicine Grant Support (20161425), Shanghai Jiaotong University Medical Cross Fund (YG2017MS28), Shanghai Municipal Science and Technology Committee (14411966800), and the Techpool Fund (UF201419). No funders have any roles in study design, data collection and analysis, decision to publish, or preparation of the manuscript.

## Conflict of Interest

The authors declare that the research was conducted in the absence of any commercial or financial relationships that could be construed as a potential conflict of interest.
