# Supplementary material for: Aberrant Non-Coding RNA Expressed in Gastric Cancer and Its Diagnostic Value
Source: Front Oncol. 2021 Jul 6;11:606764. doi: 10.3389/fonc.2021.606764 (PMC8291998; doi:10.3389/fonc.2021.606764)

**Supplement Table 1 The primers used for real-time PCR analysis.**

| Genes | Primer sequences |
| --- | --- |
| miR-509-3-5p | forward 5’-AGTACTGCAGACGTGGCAATCATG-3’ |
| miR-550a-5p | forward 5’-CAGTGCCTGAGGGAGTAAGAGCCC-3’ |
| miR-660-5p | forward 5’-GCGCGTACCCATTGCATATCGGAGTTG-3’ |
| miR-936 | forward 5’-CACAGTAGAGGGAGGAATCGCAG-3’ |
| miR-1306-3p | forward 5’-CACGTTGGCTCTGGTGGTG-3’ |
| miR-3185 | forward 5’-AGAAGAAGGCGGTCGGTCTGCG-3’ |
| miR-6083 | forward 5’-CGCGCCTTATATCAGAGGCTGTGGG-3’ |
| miR-659-3p | forward 5’-CTTGGTTCAGGGAGGGTCCCCA-3’ |
| miR-6792-3p | forward 5’-CTCCTCCACAGCCCCTGCTCAT-3’ |
| U6 snRNA | forward 5'- CTCGCTTCGGCAGCACA-3' |
| External control | forward 5'- UCACCGGGUGUAAAUCAGCUUG -3’ |
| GAS5_39 | forward 5’- GGTATGGAGAGTCGGCTTGA -3' |
|  | reverse 5’- CATGCTTGCTTGTTGTGGTC -3’ |
| lnc-ABCC5-2_1 | forward 5’-GAGATGAGGCTGTTGGTGGA-3’ |
|  | reverse 5’-ATTACAGGCGTGAGCCACTG-3’ |
| lnc-RNF135-1:3 | forward 5’-CCTCAGACAGCAGTGGCATC-3’ |
|  | reverse 5’-TGCGCTGCTAACATCACTCTC-3’ |
| lnc-PSCA-4:2 | forward 5’- CAGGTGAGCAACGAGGACTG-3’ |
|  | reverse 5’-ACACAGATGCGGACAGATGG-3’ |
| lnc-MB21D1-3:5 | forward 5’- GGACAAGAAGGCTGCTGGA-3’ |
|  | reverse 5’-GACCGTTCTTCCACCACTGA-3’ |
| cicRNA-ASH2L | forward 5’-CAAGAAGGCCCGGAGGATA-3’ |
|  | reverse 5’-CCAAGTCATTTTCCCAGGTCTC-3’ |
| circRNA-MLLT10 | forward 5'- ACTTTCTGACCAGCAACGACA-3’ |
|  | reverse 5'- GCAAATGCCCAGAAGACTGC -3’ |
| circRNA-CCDC9 | forward 5’-CTGTGGCCTTGAGCACGAG-3’ |
|  | reverse 5’-CATACTCGGCGATCTCTCCA-3’ |
| circRNA-NHSL1 | forward 5’-GCCCATTCTGTGATGATTACCAA-3’ |
|  | reverse 5’-GTCCATCGGCTTTCCTCATCA-3’ |
| Human GAPDH | forward 5’-CGGATTTGGTCGTATTGGG -3’ |
|  | reverse 5’-TGGAAGATGGTGATGGGATT-3’ |

**Supplementary Figure 1**

(A-B) The expression of miR-1306-3p was negatively associated with TNM stage and lymphatic metastasis. (C-D) The expression of miR-6792-3p was positively associated with TNM stage and lymphatic metastasis. (E) The expression of lnc-PSCA-4:2 was nagetively associated with T differentiation. (F) The expression of lnc-MB21D1-3:5 was positively associated with TNM stage. (G) The expression of miR-6792-3p was positively associated with that of CA724. (H) The expression of miR-6083 was positively associated with that of CEA.

**Supplementary Figure 2**

The difference between the expression of six miRNA in plasmas and corresponding plasma exosomes.

**Supplementary Figure 3**

The top 200 TFs terms for aberrant expressed lncRNA co-expression genes bewteen GC and normal tissues.

**Supplementary Figure 1**


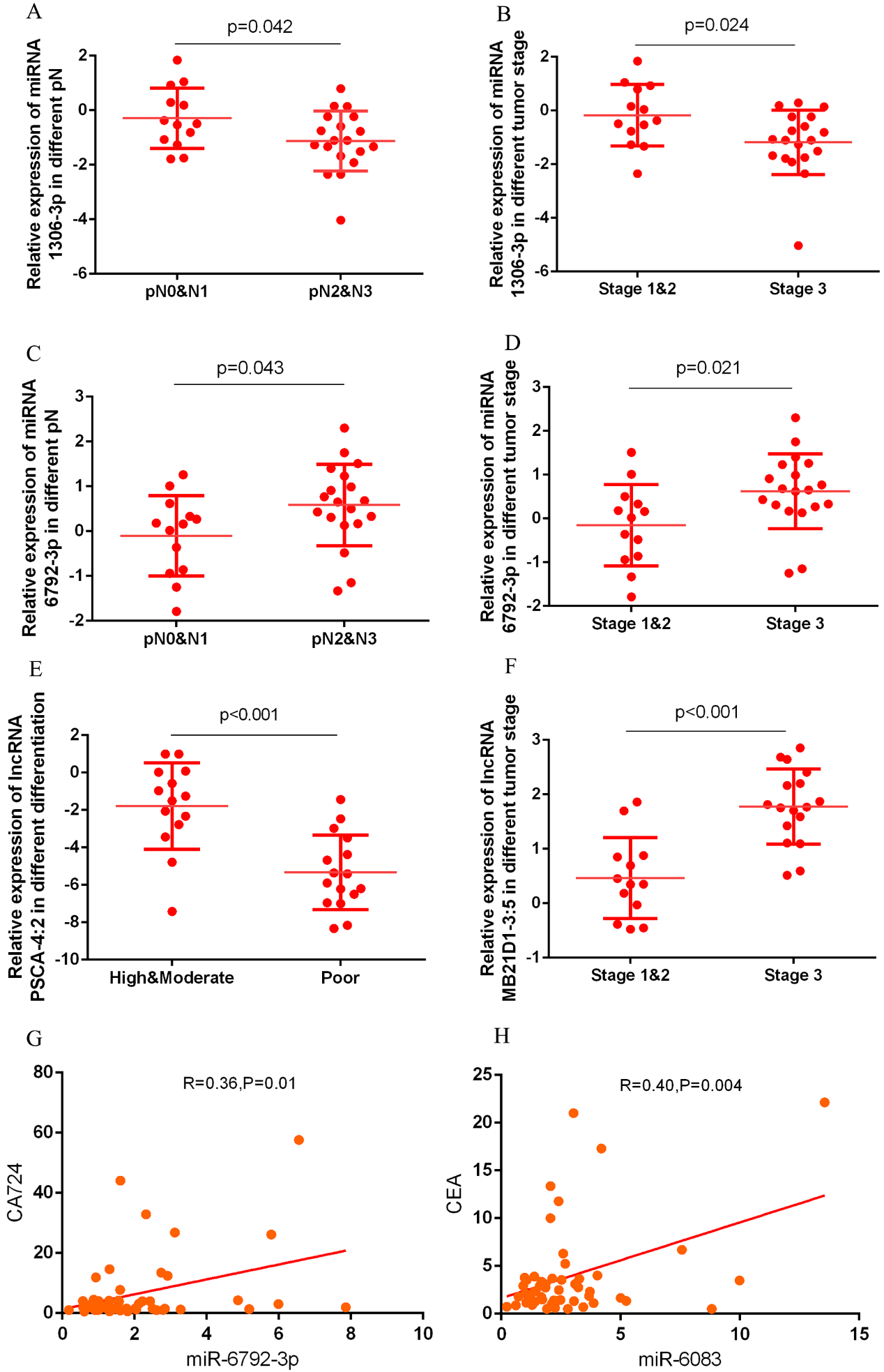


**Supplementary Figure 2**


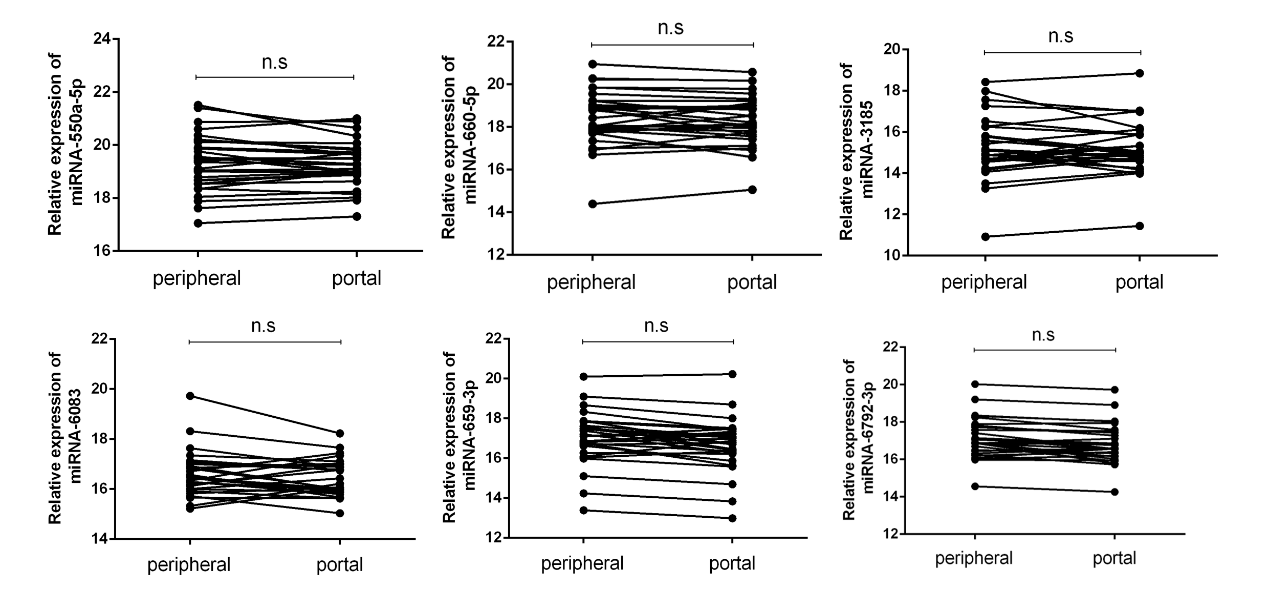


**Supplementary Figure 3**


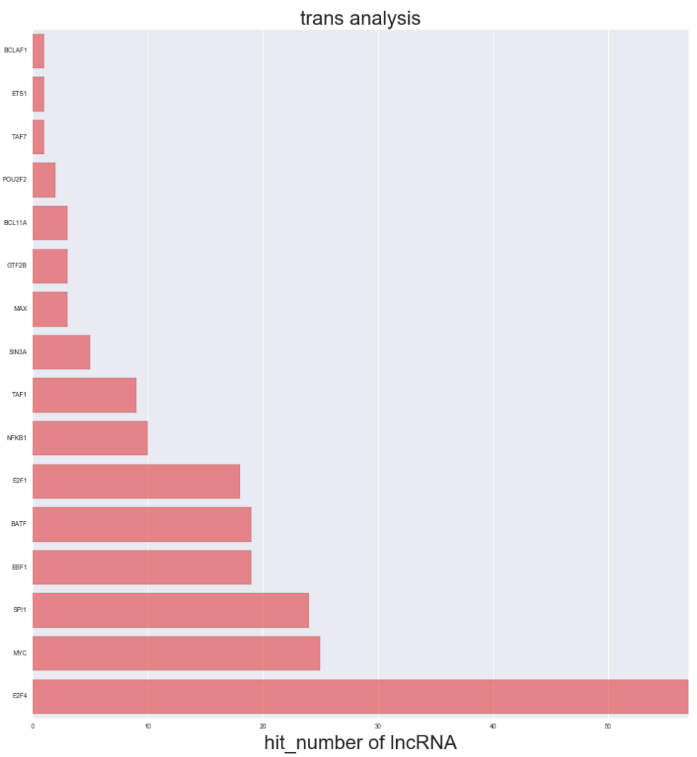

Supplement: Supplementary file 1 [file DataSheet_1.docx]
